# Hydroxyapatite from Natural Sources for Medical Applications

**DOI:** 10.3390/ma15155091

**Published:** 2022-07-22

**Authors:** Laura Madalina Cursaru, Miruna Iota, Roxana Mioara Piticescu, Daniela Tarnita, Sorin Vasile Savu, Ionel Dănuț Savu, Gabriela Dumitrescu, Diana Popescu, Radu-Gabriel Hertzog, Mihaela Calin

**Affiliations:** 1 National R&D Institute for Non-Ferrous and Rare Metals, INCDMNR-IMNR, 102 Biruintei Blvd, 077145 Pantelimon, Romania; iota.miruna@imnr.ro (M.I.); micalin@inoe.inoe.ro (M.C.); 2 Department of Applied Mechanics, Faculty of Mechanics, University of Craiova, 200585 Craiova, Romania; daniela.tarnita@edu.ucv.ro; 3 Department of Engineering and Management of Technological Systems, Faculty of Mechanics, University of Craiova, 200585 Craiova, Romania; sorin.savu@edu.ucv.ro (S.V.S.); ionel.savu@edu.ucv.ro (I.D.S.); 4 “Cantacuzino” National Military Medical Institute for Research and Development, Splaiul Independenței nr. 103, Sector 5, 050096 Bucharest, Romania; dumitrescu.gabriela@cantacuzino.ro (G.D.); popescu.diana@cantacuzino.ro (D.P.); raduhg@yahoo.co.uk (R.-G.H.); 5 National Institute of Research and Development for Optoelectronics INOE 2000, 409 Atomistilor Street, 077125 Magurele, Romania

**Keywords:** hydroxyapatite, nano-crystalline powders, hydrothermal synthesis, mechanical properties, cell viability, cell proliferation

## Abstract

The aim of this work is to study the physical-chemical, mechanical, and biocompatible properties of hydroxyapatite obtained by hydrothermal synthesis, at relatively low temperatures and high pressures, starting from natural sources (Rapana whelk shells), knowing that these properties influence the behavior of nanostructured materials in cells or tissues. Thus, hydroxyapatite nanopowders were characterized by chemical analysis, Fourier-transform infrared spectroscopy (FT-IR), dynamic light scattering (DLS), scanning electron microscopy (SEM), and X-ray diffraction (XRD). In vitro studies on osteoblast cell lines (cytotoxicity and cell proliferation), as well as preliminary mechanical tests, have been performed. The results showed that the obtained powders have a crystallite size below 50 nm and particle size less than 100 nm, demonstrating that hydrothermal synthesis led to hydroxyapatite nanocrystalline powders, with a Ca:P ratio close to the stoichiometric ratio and a controlled morphology (spherical particle aggregates). The tensile strength of HAp samples sintered at 1100 °C/90 min varies between 37.6–39.1 N/mm^2^. HAp samples sintered at 1300 °C/120 min provide better results for the investigated mechanical properties. The coefficient of friction has an appropriate value for biomechanical applications. The results of cell viability showed that the cytotoxic effect is low for all tested samples. Better cell proliferation is observed for osteoblasts grown on square samples.

## 1. Introduction

Hydroxyapatite (HAp) is a well-known calcium phosphate material, chemically identical to the mineral phase of the bone and the hard tissues of mammals. The most interesting property of this ceramic material is its ability to interact with living bone tissue, forming strong bonds with the bone, without causing toxicity or inflammatory response. It is commonly used for orthopedic, dental, and maxillofacial applications, either as a coating material for metal implants or as a bone filler. However, the material has some disadvantages. HAp is not thermally stable, with dehydroxylation starting at 800–1200 °C, depending on its stoichiometry [1,2]. It has poor mechanical properties (especially low fatigue strength), which means that it cannot be used in compact form for applications where the implant is subjected to heavy mechanical stresses (e.g., hip joint). The mechanical properties of HAp depend on porosity, density, sinterability, crystal size, and phase composition [3]. Nanoscale hydroxyapatite crystals show better mechanical properties and greater bioactivity than micron-sized crystals [4,5].

Hydroxyapatite in various forms, such as powder, porous blocks, or pearls, can be used to fill bone defects and free spaces in the bone [6,7,8,9]; these occur when parts of the bone have been removed due to a disease (bone cancer), or when bone extensions are needed (in the case of dental applications). The bone filling will form a skeleton and will facilitate the rapid filling of the pores by the growing natural bone tissue [2]. Hydroxyapatite as a filler is an alternative to autologous bone grafts, becoming part of the bone structure and reducing the time required to heal diseased tissue [10].

Recently, hydroxyapatite has been studied for other applications such as drug-delivery [11,12,13], collagen stimulation [14], skin regeneration [15,16], or sun protection in the pharmaceutical industry [15], as well as water purification [17,18,19,20], wastewater treatment [21,22,23,24], and in other chemical, optical, and electronics industries [1].

Therefore, various methods for synthesizing Hap, with tailored properties, have been investigated. These can be classified as dry methods (solid-state and mechanochemical), wet methods (chemical precipitation, hydrolysis, sol-gel, hydrothermal, emulsion, polymer-assisted routing, synthesis via biological tissue, ultrasonic spray freeze-drying, microwave irradiation, and sonochemical procedures), and high temperature processes (combustion and pyrolysis) [25,26,27,28,29,30].

Although many synthesis methods have been developed, the preparation of HAp with specific characteristics remains challenging because of the possibility of the formation of toxic intermediary products or impurities during the synthesis of HAp [5]. Thus, studies on new synthesis parameters of HAp are still in progress [27].

In recent years, many researchers combined the synthesis methods of HAp with the sustainable use of CaCO_3_ natural resources, namely the processing of marine (seashells, fish bone, corals, algae) and agricultural wastes (eggshells, animal bones) for preparing calcium phosphates [31,32,33,34].

Compared to synthetic HAp, natural HAp is non-stoichiometric, containing traces of Na^+^, Zn^2+^, Mg^2+^, K^+^, Si^2+^, Ba^2+^, F^−^, and (CO_3_)^2−^, which resembles the chemical composition of human bone [35,36].

In the present study, our goal is to study the physical-chemical, mechanical, and biocompatible properties of hydroxyapatite prepared by the hydrothermal method in different pressure conditions, starting from Rapana whelk shells from the Black Sea coast, knowing that these properties influence the behavior of nanostructured materials in cells or tissues. As a novelty, the influence of synthesis pressure on the physical-chemical properties of HAp is studied, aiming to prepare highly crystalline HAp with improved mechanical and biocompatible properties.

## 2. Materials and Methods

### 2.1. Materials

Rapana Thomasiana shells were collected from the Romanian Black Sea coast, cleaned of sand, and washed with water and detergent to remove algae and traces of visceral mass inside. The commercial materials used were NH_4_H_2_PO_4_ p.a. (Lach-Ner, s.r.o., Neratovice, Czech Republic), HCl 37% p.a. (Cristal R Chim SRL, Bucharest, Romania), HNO_3_ 68% p.a. (Cristal R Chim SRL, Bucharest, Romania) and NH_3_ 25% p.a. (Cristal R Chim SRL, Bucharest, Romania).

### 2.2. Hydrothermal Synthesis

Prior to hydrothermal synthesis, Rapana Thomasiana shells were mechanically crushed, ground in a Retsch Vibratory Disc Mill RS 200 (Retsch GmbH, Haan, Germany), and dissolved in a mixture of HCl:HNO_3_ = 2:1 (60 mL HCl 37% and 30 mL HNO_3_ 68%), resulting in a solution of 30–40% calcium (Ca precursor of HAp). Afterwards, this solution was magnetically mixed with NH_4_H_2_PO_4_ as the phosphorus precursor, precipitated with NH_3_ solution 25% until alkaline pH 10, and subjected to hydrothermal synthesis in a Teflon vessel placed in a closed system (Berghof reactor, Berghof Products + Instruments, GmbH, Eningen unter Achalm, Germany). The hydrothermal process was conducted at temperatures between 100–150 °C and various pressures (2, 6, and 10 MPa, respectively), followed by drying in a Memmert oven UFE 400 (MEMMERT GmbH + Co. KG, Schwabach, Germany) at 100 °C for 24 h. The pressure was created by bubbling Ar 5.0 gas (99.999% purity) over the aqueous solution in the Teflon vessel.

### 2.3. Characterization of Hydroxyapatite Powder

The characterization of the nanostructured HAp powder was performed by the following methods: flame atomic absorption spectrometry (FAAS), for the determination of Ca content; inductively coupled plasma optical emission spectrometry (ICP-OES), to determine P content; Fourier transform infrared spectroscopy (FT-IR), to highlight the vibrational modes; X-ray powder diffraction (XRD), for phase analysis and crystallite size determination; Dynamic light scattering (DLS), for particle size distribution in suspension; BET specific surface area determination; scanning electron microscopy (SEM), coupled with energy dispersive X-ray spectroscopy (EDX), for morphology analysis; and differential scanning calorimetry (DSC), coupled with thermogravimetry (TGA), for thermal stability.

#### 2.3.1. Chemical Analysis

FAAS was performed using an Analytik Jena ZEEnit 700 P AAS Atomic Absorption Spectrometer (Jena, Germany). For ICP-OES analysis, an Agilent 725 ICP-OES system (Agilent Technologies, Santa Clara, CA, USA) was used.

#### 2.3.2. Structural Analysis

The presence of functional groups characteristic of hydroxyapatite was identified by FT-IR, using an ABB MB 3000 FT-IR spectrometer (ABB Inc., Québec, QC, Canada), equipped with the EasiDiff device (PIKE Technologies, Inc., Madison, WI, USA) for working with powders. Measurements were conducted in the transmission mode, from 4000 to 550 cm^−1^, with a scan resolution of 4 cm^−1^. Experimental data were processed using the Horizon MBTM FTIR software version 3.4.0.3 (ABB Inc., Québec, QC, Canada).

In the case of X-ray diffraction, the data acquisition was performed on the BRUKER D8 ADVANCE diffractometer (Bruker AXS GmbH, Karlsruhe, Germany) using the DIFFRAC plus XRD Commander software, version 5.1.0.5 (32 Bit) Bruker AXS 2010-2019 (Bruker AXS GmbH, Karlsruhe, Germany), according to the Bragg-Brentano diffraction method, θ-θ coupling in vertical configuration, at a voltage of 40 kV and current of 40 mA, in the range 2θ = 4 ÷ 74°, and 2θ step of 0.03°. The phase identification was completed with the help of the DIFFRAC.EVA release 2019 program (Bruker AXS GmbH, Karlsruhe, Germany) from the DIFFRAC.SUITE.EVA software package and the ICDD PDF4 + 2022 database.

#### 2.3.3. Particle Size Distribution

Particle size distribution was measured using a Zetasizer Nano ZS 90 particle analyzer, Malvern Instruments (Worcestershire, UK), in size range of 0.3–5.0 µm, temperature range of 2–90 °C, and endowed with Zetasizer software v8.01 (PSS0012-42, Malvern Instruments Ltd., Malvern Panalytical Ltd., Worcestershire, UK).

Sample preparation: a stable suspension was prepared by magnetic stirring and the sonication of nanostructured HAp powder with double distilled water, ethanol, and commercial dispersant Duramax^TM^ D3005 (Trademark of The Dow Chemical Company, Midland, MI, USA). The obtained suspension was filtered through a Millipore membrane (d = 0.22 μm) and transferred to the glass cuvette for particle size distribution measurement.

#### 2.3.4. BET Specific Surface Area Measurements

The method used to determine specific surface area, pore volume or porosity, and pore shape and size is based on the physisorption of N_2_ gas at 77 K (−196 °C), with an adsorption-desorption isotherm. Measurements were performed using a Micromeritics TriStar II Plus analyzer (Micromeritics Instrument Corporation, Norcross, GA, USA). The specific surface area was obtained by the Brunauer–Emmett–Teller (BET) method, while the pore volume and pore size distribution were determined by the Barrett–Joyner– Halenda (BJH) method. Prior to each determination, the powder samples were subjected to a heat treatment at 300 °C for several hours to remove traces of liquids and impurities using the VacPrep 061 degassing stations.

#### 2.3.5. Morphological Analysis

To examine and correctly establish the morphology and size of the hydroxyapatite crystals obtained, the hydroxyapatite samples were studied in High Vacuum mode using a FEI Quanta 250 scanning electron microscope (FEI Company, Eindhoven, The Netherlands). The analyzed samples were metallized by coating with a 5 nm thick Au layer.

### 2.4. Hydroxyapatite Pellets Preparation

HAp nanopowders prepared by hydrothermal synthesis at 10 MPa were mechanically mixed with polyvinyl alcohol (PVA) 5% solution, dried in an oven at 100 °C for 24 h, and then uniaxially compacted at a pressure of 98 MPa into cylindrical pellets with a 16 mm diameter and a 12 mm height for mechanical evaluation, respectively, and round disks with a diameter of 9 mm and a height of 1.6 mm for in vitro testing. These pellets were further sintered in air atmosphere at different temperatures and sintering times (1100 °C/90 min; 1200 °C/90 min, 120 min, 180 min; 1300 °C/90 min, 120 min, 180 min; and 1400 °C/90 min, 120 min, 180 min, respectively) in an electric furnace at a heating and cooling rate of 1 °C/min and allowed to furnace cool. Sintering conditions were chosen based on literature data presented in [37,38]. HAp sintered specimens are presented in Table 1.

### 2.5. Mechanical Properties Evaluation

To investigate the mechanical properties of the sintered specimens, all tests (compressive strength evaluation, micro-hardness measurement, and wear test) have been conducted in accordance with the international standards in force [39,40], but also based on our previous experience [41,42].

#### 2.5.1. Compressive Strength Evaluation

The universal test machine LBG 100 (Maximum force: 100 kN) was used for the compression testing (performed according to our own procedure, based on EN 658-2:2002).

#### 2.5.2. Micro-Hardness Measurement

The micro hardness (HV1) of the sintered specimens was determined according to ISO 14705:2000, via the Vickers indentation, with a NAMICON CV-400DM Microdurimeter produced by CV Instruments Europe BV (range: HV0,01-HV1, load 10–1000 g, resolution 0.03 µm, Vickers diamond indenter, 10×, 40× objectives, microscope with analog reading, automatic load force control, video image control). A total of 3 indentations were made and the resulting hardness values were averaged.

#### 2.5.3. Wear Test

A CSM Instruments tribometer, with a maximum torque of 450 N.mm and a maximum load of 46 N., was used for the wear tests. The software used for analysis and graphical representation was InstrumX. The usable frequency is 1.6 Hz at a speed of ball movement in the range of 0.3–500 mm/s, the frequency of information acquisition being 10 Hz. The tests were performed according to our own developed procedure, based on ISO 22622:2019, with harder conditions (100 Hz instead 10 Hz).

### 2.6. Biocompatibility Assessment

#### 2.6.1. Preparation of Cell Lines

To perform the cytotoxicity test of the HAp samples, a cell line of normal human osteoblasts (NHOst, Cat.No. CC-2538, Lonza, Germany) was used. The osteoblast cell line was cultured in a 25 cm^2^ cell culture flask using osteoblast-specific growth medium, supplemented with 10% fetal bovine serum (FBS) and 50 μg/mL gentamicin. Cells from two flasks of 25 cm^2^ cells were trypsinized with 0.025% trypsin-EDTA, centrifuged, and the pellet was resuspended in 5 mL of growth medium completely specific to the cell line. Initial cell counting was performed by staining with Tripan Blue 1:1 (50 μL cell suspension + 50 μL Tripan Blue). The osteoblast cell suspension has a concentration of 1.3 × 10^6^ cells/mL.

#### 2.6.2. Preparation of the Test Compound

A total of 12 HAp samples were assessed for cytotoxicity and cellular proliferation: 6 square samples, with dimensions of 15 × 15 × 5 mm^3^, fabricated using the 3D printing technique as described in [43], dried in oven at 100 °C, and not sintered; and 6 round samples (disks), with a diameter of 9 mm and a height of 1.6 mm, sintered at 1200 °C/180 min, as shown in Table 1.

The 12 tested samples were placed in a 12-well cell culture plate, with growth medium completely specific to the osteoblast cell line (OGM^TM^ Osteoblast Growth medium BulletKit^TM^, Lonza, Germany), and incubated for 24 h at 37 °C, in a shaking incubator, 5% CO_2_. The weight/volume ratio was 200 mg/mL, according to the recommendations of ISO 10993-12. After incubation, the stock solution was diluted in binary dilutions (1/2, 1/4, and 1/8).

#### 2.6.3. Cytotoxicity Test

To perform the cytotoxicity test, the osteoblast cell suspension is cultured in 2 microplates with 96 wells and a flat bottom, 200 μL/well (2.6 × 10^5^ cells/well), and incubated in a CO_2_ incubator (5%) at 37 °C for 24 h.

The next day, the environment is changed, as follows:

- Only 200 μL of complete growth medium is added to the blank wells.
- In the cell control wells, the medium is removed, and 200 μL of complete, fresh growth medium is added.
- The medium is removed from the wells with the test compound, and 200 μL of fresh medium containing various dilutions of the compound (1/2, 1/4, and 1/8) are added. The blank, the cell control, and the test samples are distributed in duplicate for each dilution.

After 24 h, the 3-4,5-(dimethyl-2-thiazolyl)-2,5-diphenyl-2H-tetrazolium bromide (MTT) test is performed to measure the conversion of MTT to a stained product in living cells. The MTT-based cell growth assay kit (Sigma-Aldrich, Darmstadt, Germany) containing the MTT solution (5 mg/mL MTT in RPMI-1640 without phenol red) and the MTT solvent (0.1 N HCl in anhydrous isopropanol) were used for this assay. The microplates are removed from the incubator, 20 μL MTT/well (10% of the medium volume) is added, and the microplates are incubated for 4 h at 37 °C, in dark, under CO_2_. After 4 h, the microplates are removed from the incubator, the medium is discarded, and 200 μL of MTT solution/well is added. The optical density (OD) is read at a wavelength of 570 nm, within a maximum of 1 hour from the time the solvent was added, using a multimodal reader (EnSight™ Multimode Microplate Reader, PerkinElmer, Akron, OH, USA).

Cell viability is calculated with the following formula:

$$\%\;\mathrm{Cell}\;\mathrm{viability}\;=\frac{\mathrm{OD}\;\mathrm{positive}\;\mathrm{control}\;-\;\mathrm{OD}\;\mathrm{blank}}{\mathrm{OD}\;\mathrm{negative}\;\mathrm{control}\;-\;\mathrm{OD}\;\mathrm{blank}}\times 100$$

where: positive control = cells + compound + MTT + MTT solution; negative control = cells + MTT + solvent MTT; and blank = complete growth medium + MTT + MTT solution.

The negative control (cell control) and the samples (positive control) were run in 2 wells for each concentration of the compounds and at the end, the arithmetic mean of the optical density readings was made, and read at a wavelength of 570 nm.

#### 2.6.4. Cell Proliferation

To highlight cell proliferation, the 12 samples to be tested were placed in the center of the wells of a 12-well plate and incubated with 400 µL growth medium in an incubator at 37 ° C overnight. In addition, 200 µL of osteoblast cell suspension were added, the samples being thus cultured in a 5% CO_2_ atmosphere at 37 °C, allowing the cells to be attached to the samples. A modified MTT test was used [1].

Briefly, the osteoblast cell line was seeded at a density of 2.6 × 10^4^/mL, and the cell culture was for 24, 48, and 72 h. The growth medium was replaced daily during testing.

A total of 60 µL MTT solution (5 mg/mL) was added to the wells, followed by an incubation period at 37 °C for 4 h for the formation of MTT formazan. The supernatant was removed by aspiration, and the MTT solvent (600 µL DMSO) was added to dissolve the formazan crystals. Within a maximum of one hour after the addition of the solvent, 200 µL of each well were then transferred to a 96-well plate (in duplicate) to read the optical density at a wavelength of 570 nm, using the multimodal reader (EnSight™ Multimode Microplate Reader, PerkinElmer, Hopkinton, Massachusetts, USA). For the resumption of samples in 96-well plates, the same sample arrangement schemes, with the 2 adhered cell lines but at different contact times (at 24, 48, and 72 h), were used. The interpretation of the results is made using the previously mentioned formula to determine the cytotoxicity. The obtained values reflect cell proliferation in each well, for each sample [2].

## 3. Results

### 3.1. Hydrothermal Synthesis

Hydroxyapatite nanopowders prepared from natural sources were synthesized under hydrothermal conditions at temperatures, between 100–150 °C, and different pressures (2, 6, and 10 MPa). The resulting powders were denoted HAP-20, HAP-60, and HAP-100, with the numbers in the sample code signifying the working pressure in bar units.

The chemical reactions that lead to hydroxyapatite, starting with Rapana Thomasiana shells, are written below:

CaCO_3_ + 2HCl = CaCl_2_ + H_2_O + CO_2_↑ (1)

CaCO_3_ + 2HNO_3_ = Ca(NO_3_)_2_ + H_2_O + CO_2_↑ (2)

10CaCl_2_ +6NH_4_·H_2_PO_4_ + 14NH_4_OH = Ca_10_(PO_4_)_6_OH_2_ + 20NH_4_Cl + 12H_2_O (3)

10Ca(NO_3_)_2_ + 6NH_4_·H_2_PO_4_ + 14NH_4_OH = Ca_10_(PO_4_)_6_OH_2_ + 20NH_4_NO_3_ + 12H_2_O (4)

Equations (1) and (2) describe chemical reactions which take place duringthe dissolving of Rapana shells in the HCl-HNO_3_ mixture. Hydrothermal reactions are represented by Equations (3) and (4). Calcium solution, consisting of CaCl_2_ and Ca(NO_3_)_2_ aqueous species, is mixed with NH_4_·H_2_PO_4_ as the P precursor of HAp and precipitated with NH_3_ 25% solution. During hydrothermal synthesis at a high temperature and pressure, crystalline HAp nanoparticles are formed.

It is well known that crystalline hydroxyapatite can be obtained using the hydrothermal method in a relatively wide temperature range (from 70 °C to 200 °C) [4,44,45,46]. An important advantage of using the hydrothermal process for HAp synthesis, besides controlled morphology and nanometer particle size, is that no hydroxyl defects are produced in the structure [44].

Hydrothermal synthesis takes place in a perfectly sealed reaction system, in aqueous solution, at a high temperature and pressure. Usually, pressure is created by the saturated vapor phase which forms above the solution, and the most varied hydrothermal synthesis parameters, according to literature data, are temperature, time, and pH [44,46]. Although preliminary results on hydrothermal synthesis of HAp obtained from Rapana shells in high pressure conditions (10 MPa) were reported in our previous paper [43], the effect of pressure on the physical-chemical properties of HAp was not investigated.

Numerous scientific papers [47,48,49,50] have developed various models for thermodynamic calculation of physicochemical processes that take place in aqueous or non-aqueous solutions during hydrothermal synthesis. Depending on the results obtained, not only can the appropriate solvent be selected, but also the pressure-temperature range that leads to the formation of the desired reaction products and allows for the control of the shape and size of the obtained particles. The behavior of the solvent under hydrothermal conditions, in terms of its structure in critical, super-critical, and sub-critical conditions, as well as the dielectric constant, pH variation, viscosity, coefficient of expansion, and density, must all be correlated with the temperature and working pressure.

In the case of high-pressure hydrothermal synthesis, an external pressure higher than the water vapor pressure at equilibrium is used. Under these conditions, remarkable results are obtained because the solubility of inorganic materials increases with increasing pressure. Thermodynamic stability also varies with pressure (at very high pressures, denser phases crystallize). Hydrothermal reactions are based on the equilibrium reactions of dissolution-reprecipitation and crystallization.

In the studied system, the pressure above the aqueous suspension in the autoclave vessel corresponds to the vapor pressure of the substances in the reaction system, but also to the external pressure of the inert gas introduced. The temperature is kept constant at values much lower than the critical water temperature (T = 100–150 °C << 374 °C) so that the liquid–gas equilibria will be neglected. In this way, due to the high pressure in the reaction system, the crystallization of hydroxyapatite under hydrothermal conditions takes place at temperatures much lower than the usual values for obtaining crystalline calcium phosphates. The influence of the pressure on the equilibrium constant for real systems is determined by the variation of the molar volumes of the participants in the reaction (reactants and reaction products), denoted Δ^r^V. In the case of hydrothermal reactions Δ^r^V < 0, the value of the equilibrium constant increases with increasing pressure, meaning the equilibrium shifts to the reaction products (HAp formation reaction is favored by the introduction of external pressure). The introduction of external pressure in the hydrothermal synthesis autoclave favors the obtaining of nanostructured crystalline HAp at relatively low temperatures.

The chemical analysis results (Ca:P ratio) are shown in Table 2.

The Ca:P ratio is higher than theoretical value of Ca:P = 1.67, which is calculated from the HAp chemical formula Ca_10_(PO_4_)_6_(OH)_2_, which could be explained by the formation of non-stoichiometric hydroxyapatite, because of the natural Ca source used in the synthesis.

### 3.2. Structural Analysis

#### 3.2.1. FT-IR Analysis

**Figure 1:**
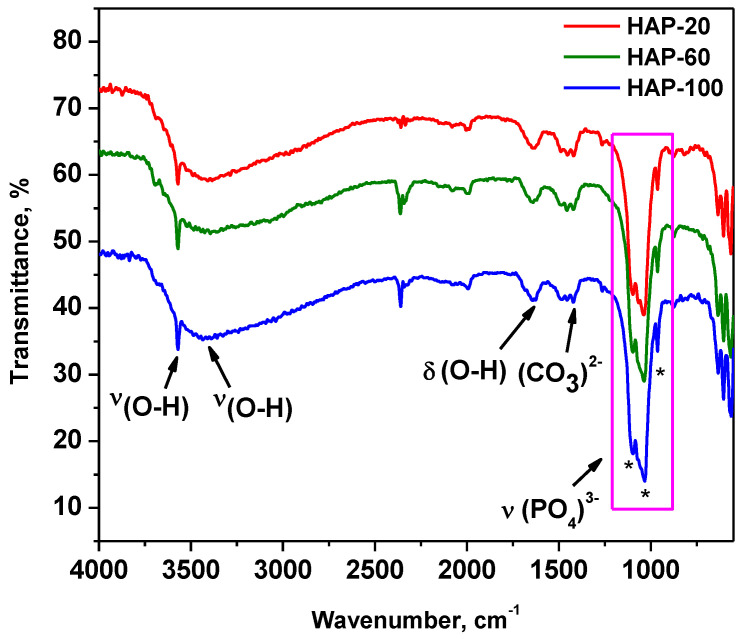
FT-IR spectra of HAp nanopowders.

Figure 1 shows the superposed FT-IR spectra of the hydroxyapatite nanopowders prepared at different pressures (2, 6, and 10 MPa, respectively). The characterization of HAp nanostructured powders by FT-IR highlighted the presence of the following vibration bands for all the investigated samples: (i) the stretching vibration of the OH group (sharp band) from 3570 cm^−1^; (ii) the stretching vibration of the H_2_O molecule (broad band) from 3250–3500 cm^−1^ [25]; (iii) the deformation vibration of the OH group (1641–1651 cm^−1^), associated with the stretching vibrations from 3250–3500 cm^−1^; and (iv) the stretching vibrations of the (PO_4_)^3−^ group from 1095–1097 cm^−1^, 1032–1038 cm^−1^, and 962 cm^−1^, respectively, characteristic of hydroxyapatite [51]. The medium intensity bands in the range of 1420–1489 cm^−1^ are due to the (CO_3_)^2−^ group [52].

Comparing the absorbance ratios A_1097_/A_1487_ and A_962_/A_1421_ of the peaks characteristic of (PO_4_)^3−^ (1097 and 962 cm^−1^) to those characteristic of (CO_3_)^2−^ (1487 and 1421 cm^−1^), at different pressures, provides information regarding the amount of hydroxyapatite in the nanopowder samples [53]. The values of the peak area ratios are displayed in Table 3.

It can be observed that HAP-100 has the highest peak area ratios among the studied samples (15.25 and 1.42). These values represent the ratio between phosphate and carbonate. As a conclusion, nanopowder synthesized at 10 MPa has the highest content of HAp.

#### 3.2.2. X-ray Diffraction Characterization

**Figure 2:**
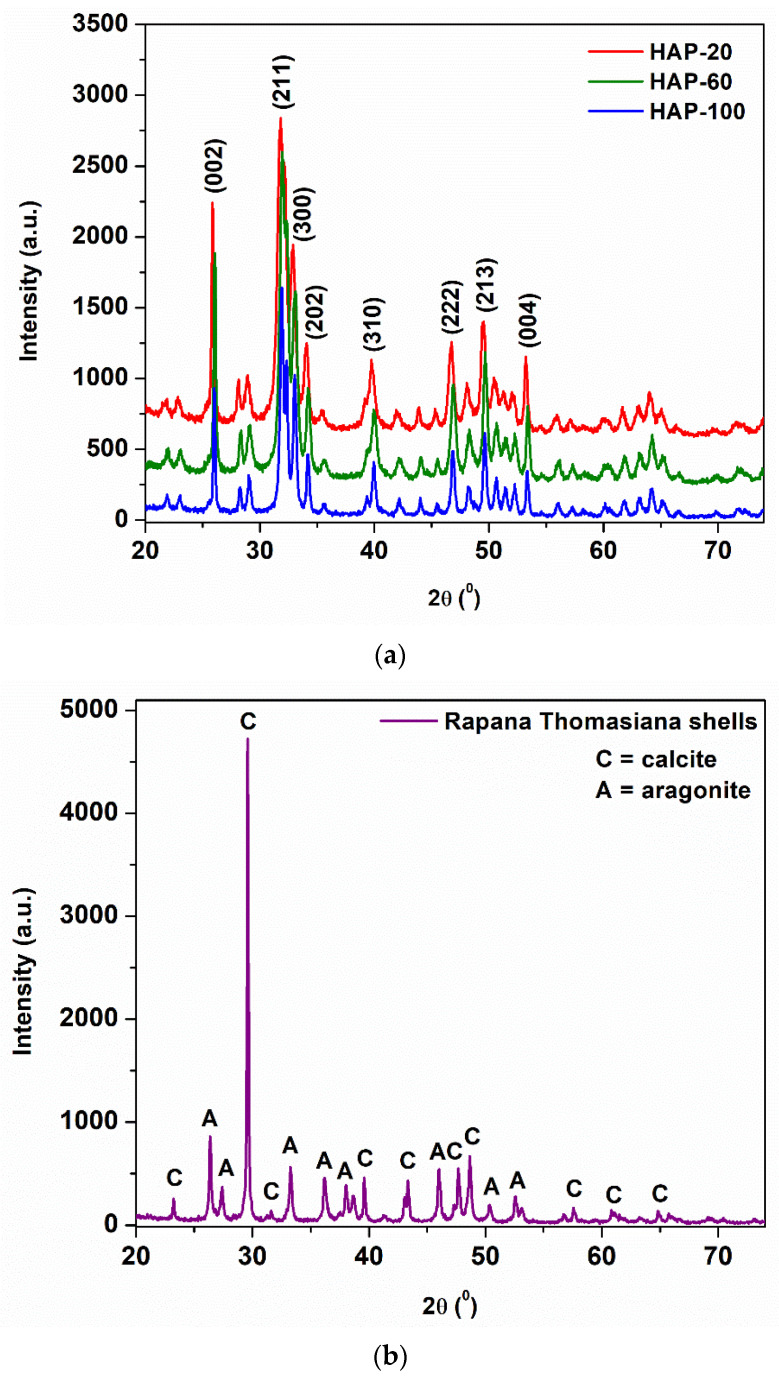
(**a**) XRD spectra of HAp nanopowders; (**b**) XRD spectrum of Rapana Thomasiana shells.

The XRD patterns of hydroxyapatite synthesized from natural sources are presented in Figure 2. For comparison, the XRD spectrum of Rapana Thomasiana shells is shown in Figure 2b. The main crystalline phases identified in these shells are calcite (chemical formula CaCO_3_, PDF reference 01-083-3288), representing~64.4% in weight, and aragonite (chemical formula CaCO_3_, PDF reference 01-075-9982), representing~35.6% in weight. The main crystalline phase identified in the powders obtained by the hydrothermal process is Hydroxylapatite, PDF reference 00-009-0432, with typical (h k l) Miller indices (002), (211), (300), (202), (310), (222), and (213). The (002) reflection peak from the XRD pattern was used to calculate the crystallite size of HAp nanopowders, using the Debye–Scherrer equation. The average particle size, measured using a BET analyzer, as well as the hydrodynamic diameter (d(H)), determined by the DLS method and the polydispersity index (PdI), are summarized in Table 4, along with the crystallite size.

The crystallinity index (CI), defined as the ratio between the total area of the narrow diffraction maxima, due to the crystalline phases, and the total diffracted area of the sample, after removing the background contribution [54,55], was determined for the three samples using the following equation:

$$\mathrm{Crystallinity}\;\mathrm{Index}\;=\frac{\mathrm{Crystalline}\;\mathrm{phase}\;\mathrm{area}}{\mathrm{Crystalline}\;\mathrm{phase}\;\mathrm{area}+\mathrm{Amorphous}\;\mathrm{phase}\;\mathrm{area}}$$

It can be observed that the dimensions of HAp nanopowders increase with increasing pressure, regardless of the type and method used for determining them (Scherrer crystallite size, BET average particle size, hydrodynamic diameter). Pressure favors the crystallites growth during hydrothermal synthesis, leading to the formation of crystalline phases with a high degree of crystallinity. The crystallinity index also increases with pressure increase, from 71% to 79%. Based on these observations, correlated with peak area ratio calculated from FT-IR spectra, HAP-100 (sample synthesized at 10 MPa) was selected for further study of the mechanical properties.

### 3.3. Analysis of Particle Size Distribution by DLS Technique

Dynamic light scattering (DLS) is an established measurement technique for the characterization of particle sizes in suspension, based on the Brownian motion of particles. The smaller the particles, the faster they will move in a solution. The hydrodynamic diameter represents the particle size plus the dielectric layer, which adheres to its surface during movement through the liquid medium. The movement of the particles causes intensity fluctuations in the scattered light. From these fluctuations, the diffusion coefficient can be determined, and thus the hydrodynamic diameter of the particle is obtained from the Stokes–Einstein equation:

$$d\left(H\right)=\frac{\mathrm{kT}}{3\pi \eta D}$$

where: d(H) = hydrodynamic diameter, k = Boltzmann’s constant (1.38 × 10^−23^ NmK^−1^), T = absolute temperature (K), η = solvent viscosity (N·s·m^−2^), and D = diffusion coefficient (m^2^·s^−1^).

The results obtained for synthesized hydroxyapatite powders (hydrodynamic diameter and polydispersity index-PdI) are presented in Table 3 and Figure S1. The average particle size (hydrodynamic diameter in aqueous solutions) varies between 76 nm and 97 nm, with a monomodal size distribution. The low values of the polydispersity index suggest that the investigated samples are homogenous in size. The PdI is situated in the range of 0.009–0.087.

### 3.4. SEM Characterization

**Figure 3:**
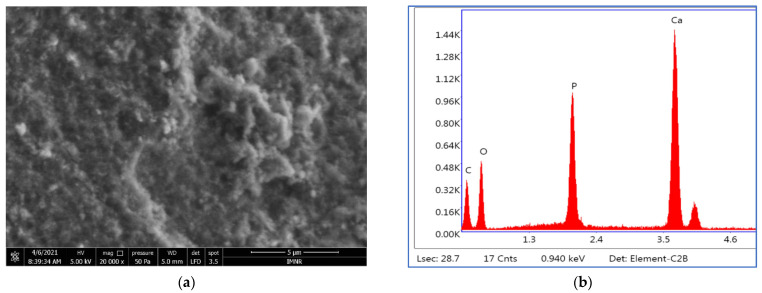
(**a**) SEM image at 5 μm scale bar, 5 kV voltage, and 20 kX magnification, and (**b**) EDS spectrum of HAP-100 nanopowder.

An SEM image of HAp nanopowder prepared at 10 MPa is presented in Figure 3.

All three analyzed samples are formed of irregularly shaped microcrystalline aggregates that have dimensions on the order of microns up to tens of microns. SEM images of HAP-20 and HAP-60 samples are presented in Figure S2. Microcrystalline aggregates are in turn formed of exceedingly small microcrystals (on the order of nanometers), whose shapes and sizes could not be highlighted. Moreover, morphological investigation revealed a porous structure of hydroxyapatite, regardless of the synthesis pressure. This porous structure, with nano-sized pores (determined by BJH method), represents an advantage for medical applications (bone tissue reconstruction) [56]. Thus, the BJH adsorption average pore width is 2.75 nm for HAP-20, 2.74 nm for HAP-60, and 2.67 nm for HAP-100 powder samples.

The EDS semiquantitative analysis results are presented in Table 5.

The Ca:P ratios calculated based on EDS analysis agree with those obtained by chemical analysis.

Based on the results obtained from the physical-chemical characterization of the HAp samples, powders prepared at 10 MPa were further selected for mechanical and in vitro testing. The reason is that HAP-100 shows the highest crystallinity index, 79.4%, calculated from XRD measurements. In X-ray diffraction, it is revealed that the smaller the crystallite size, the more amorphous the material will be considered [57]. We also assume, based on our previous results in this field, that a higher pressure positively influences the biocompatible properties of the material [43,58].

### 3.5. Mechanical Properties Evaluation

#### 3.5.1. Compressive Test Evaluation

**Figure 4:**
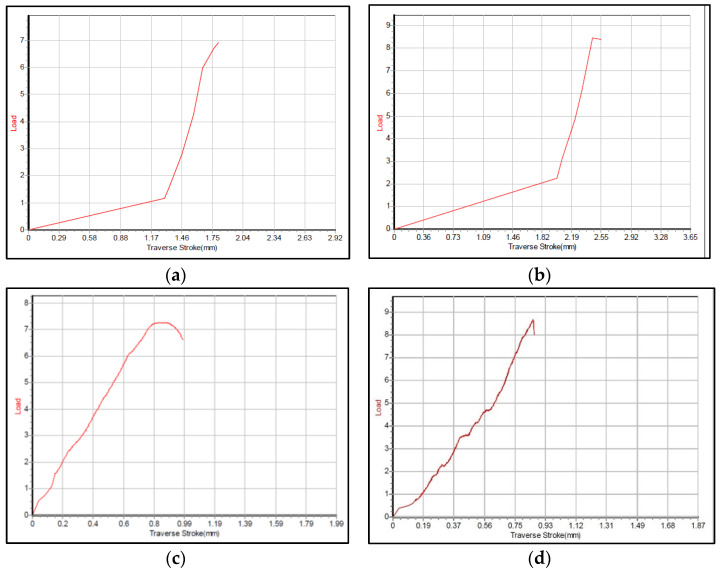
Examples of stress/strain curve for 90 min sintering: (**a**) 1100 °C/90 min; (**b**) 1200 °C/90 min; (**c**) 1300 °C/90 min; (**d**) 1400 °C/90 min.

The medical purpose application of the developed material requires appropriate compressive properties. Because of this, the compressive strengths of the tested specimens were measured, and these are presented in Table 6. Examples of strain/stress curves (for 90 min sintering time) are presented in Figure 4.

Analyzing the ranges presented in Table 6, it can be concluded that appropriate/acceptable values are obtained in the case of HAp sintered at 1200 °C/180 min, as well as at 1300 °C and 1400 °C for 120 min.

#### 3.5.2. Micro-Hardness Testing

For cylindrical specimens sintered at 1100 °C/90 min, 180 ± 5 HV1 were obtained.

**Figure 5:**
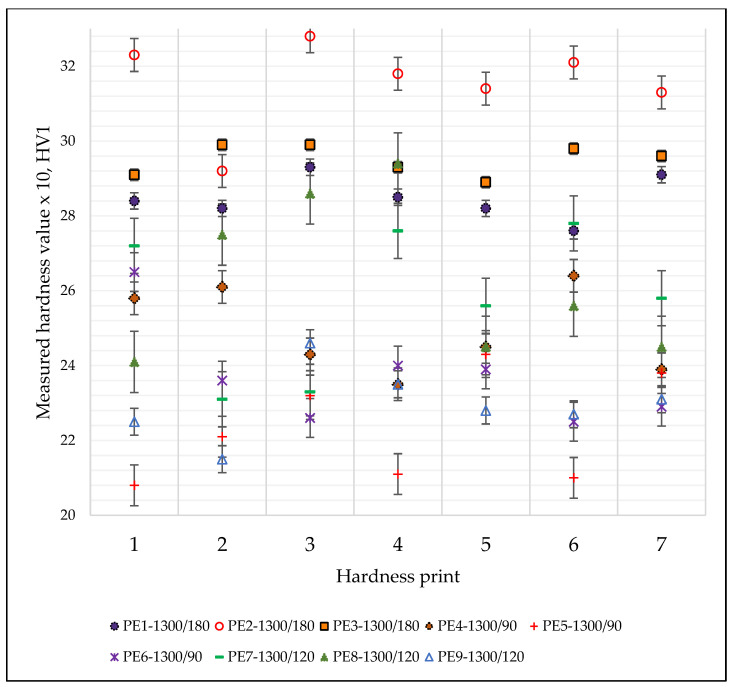
Microhardness values measured on the specimens sintered at 1300 °C.

Experimental results for the specimens sintered at 1300 °C are presented in Figure 5.

As for the tensile strength measurements, it can be said that the set of samples sintered at 1300 °C/120 min provide better results compared to specimens sintered for 90 min and 180 min, respectively.

#### 3.5.3. Wear Test

Regarding the wear test, the material sintered at 1100 °C has a low wear resistance, the fingerprint depth being 0.88 mm. Each of the other specimens that were sintered above 1100 °C showed better wear resistance compared to the values recorded for the samples sintered at 1100 °C/90 min, the fingerprint depth being less than 0.8 mm. The coefficient of friction has an appropriate value for biomechanical applications for all the investigated samples (regardless of sintering temperatures). The measured values for the sintered specimens at the four sintering temperatures are presented in Table 7.

#### 3.5.4. SEM Characterization of Sintered Specimens

**Figure 6:**
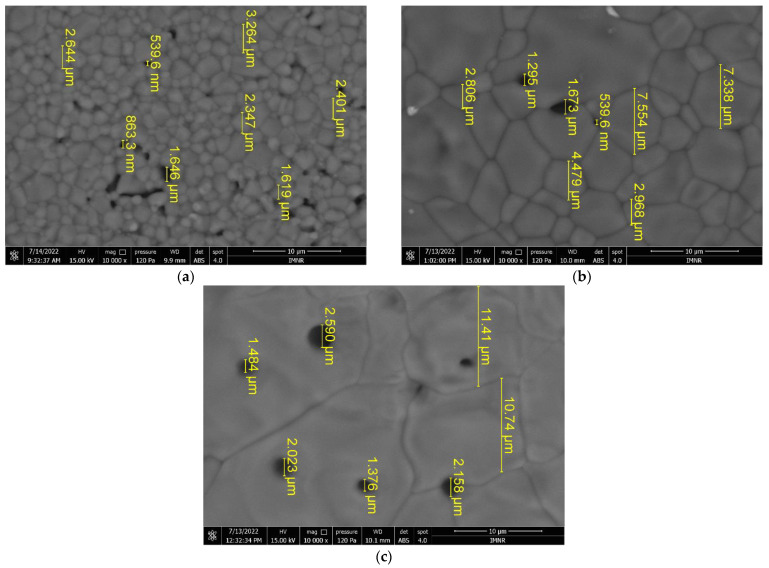
SEM images at 10 μm scale bar, 15 kV voltage, and 10 kX magnification for HAp specimens sintered at: (**a**) 1200 °C/180 min; (**b**) 1300 °C/120 min; (**c**) 1400 °C/120 min.

The sintered HAp pellets for which acceptable results were obtained from the mechanical properties evaluation were analyzed by scanning electron microscopy. Thus, SEM images of HAp specimens sintered at 1200 °C/180 min, 1300 °C/120 min, and 1400 °C/120 min, denoted as HAP-1200, HAP-1300 and HAP-1400, are shown in Figure 6.

It can be seen that the grain size increases significantly with the sintering temperature, as expected. HAP-1200 has a grain size between 1.6–3.3 µm (Figure 6a) and open porosity (pore size in the range of 539 nm–1.64 µm). A much higher degree of densification can be noticed in the case of the HAP-1300 sample (Figure 6b), with a grain size of 2.8–7.5 µm and a few pores located at the grain boundaries which are similar in size to those in the HAP-1200 sample. HAP-1400 exhibits large grains, on the order of tens of microns (10.7–11.4 µm or more) and closed porosity, with pore sizes between 1.3–2.6 µm. When the sintering temperature was increased, the porosities of the HAp specimens were reduced due to the higher densification of the material [59].

### 3.6. Biocompatibility Assessment

Biocompatibility can be broadly defined as the physical, chemical, and biological compatibility between a biomaterial and body tissues and the optimal compatibility of a biomaterial with the mechanical behavior of the body.

The biocompatibility of any biomaterial (medical device) must be evaluated using in vitro and in vivo testing before use in patients. While animal experiments are expensive and require extended periods of experimentation, cell culture methods can be performed at lower cost, are faster and easier to perform, and can be easily reproduced. In recent years, a wide range of in vitro tests have been developed to evaluate the biocompatibility of different biomaterials (powders, solutions, hydrogels, medical devices). Such an in vitro assay uses MTT {3-(4,5-dimethithiazol-2-yl)-2,5-diphenyl tetrazolium bromide} and is a sensitive, quantitative, and reliable colorimetric assay that measures cell viability, proliferation, and activation. In living cells, water-soluble yellow MTT is reduced to a dark blue formazan product by the mitochondrial dehydrogenase enzyme. The amount of formazan produced is directly proportional to the number of viable cells present. Therefore, measuring the optical density will help to determine the amount of formazan produced and thus, the number of viable cells present.

#### 3.6.1. Cytotoxicity Test

**Figure 7:**
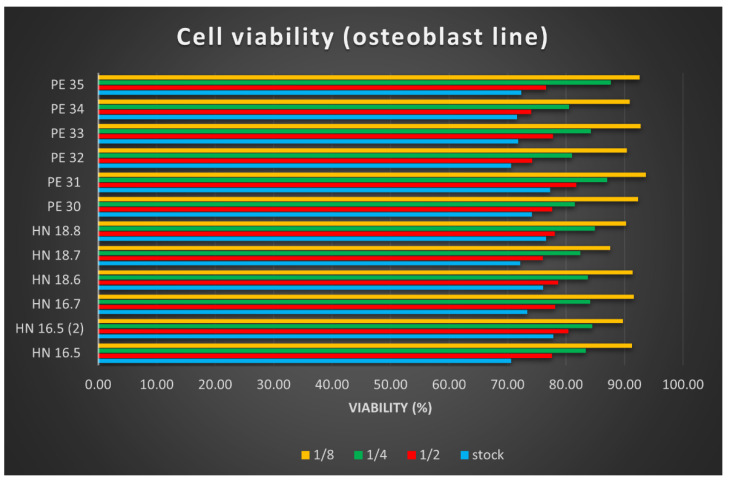
Cell viability of osteoblasts for the 12 HAp samples. The stock solution was diluted in binary dilutions (1/2, 1/4, and 1/8).

After reading the values of the optical densities for the osteoblast cell line used in testing the cytotoxicity of the 12 HAp samples, the arithmetic means of the values were calculated, and the viability calculation formula was applied. The results were plotted in Figure 7. Samples called HN-x are square specimens (3D printed samples with dimensions of 15 × 15 × 5 mm^3^), and they were studied for comparative reasons.

The cell viability results determined using the MTT test helped us to conclude the following:

- The cytotoxic effect of the 12 HAp samples tested is low, with very small differences depending on their size; square (printed) samples, dried at 100 °C showed better results.
- The cytotoxicity of the tested samples was dose-dependent; the lower the concentration of the tested product, the lower the cytotoxicity.
- The cell viability is lowest in culture wells with an extract stock concentration, and it increases in direct proportion to the increase in dilution.

#### 3.6.2. Cell Proliferation Study

**Figure 8:**
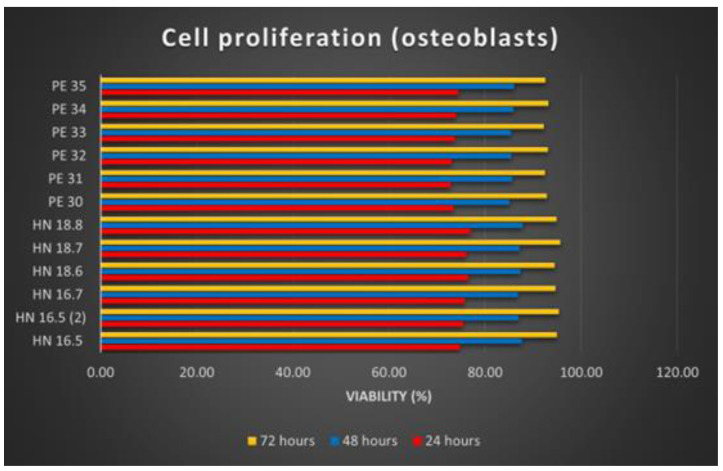
Cell proliferation of osteoblasts on HAp samples.

Osteoblast proliferation on the 12 HAp samples was analyzed at 24, 48, and 72 h of substrate–cell interaction. In the case of the cell proliferation test, after reading the absorbance values, calculating the average of the values read for each sample and applying the calculation formula, the results were synthesized as graphs (Figure 8).

The increasing number of osteoblasts used for the proliferation tests of the 12 samples shows the proliferation production on all these samples. Comparing the 12 HAp samples, it is noticeable that the differences are insignificant. Better proliferation has been observed for osteoblasts grown on square (3D printed) samples. Regarding the time dynamics of cell proliferation, it increases in direct proportion to the increase in the substrate–cell contact period.

## 4. Conclusions

In this paper, the hydroxyapatite nanopowder was obtained from natural sources of Rapana Thomasiana using hydrothermal method at various synthesis pressures. The influence of applied pressure on the physical-chemical properties of HAp powders has been explored. It was found that the crystallite size and particle size of hydroxyapatite increase with a pressure increase. The biomedical potential of the obtained material was studied through mechanical evaluation (compressive test, microhardness test, wear test), as well as cytotoxicity and cell proliferation testing. HAp specimens sintered at 1300 °C/120 min present appropriate biomechanical properties. Hardness values up to around 200 HV1 were recorded for sintering temperatures above 1200 °C and sintering times above 120 min. The compressive properties of the material returned a large range of values, from about 50 N/mm^2^ to around 120 N/mm^2^. Both the hardness and the compressive properties are larger than the values specific to bones.

Due to its function as an implant to bones, the new material is subjected to wearing. Wear tests returned nearly the same value for the coefficient of friction, which is 0.031, with penetrations between 0.68 and 0.88. The lowest penetration depths were recorded for sintering above 1200 °C and sintering times above 120 min.

Cell viability and cell proliferation increase over time. These results are encouraging, demonstrating that natural sources can be successfully exploited for the synthesis of new materials.

## Figures and Tables

**Table 1:** Sintering conditions and type of sintered for HAp specimens.

Sintering Temperature, °C	Sintering Time, Min	Sample Type	Sample Codes	Sample Destination (Mechanical Test/In Vitro Test)
1100	90	Cylinder	5.2/5.3/5.5/5.6/5.7	Mechanical
1200	90	Cylinder	PE20, PE21, PE22	Mechanical
1200	120	Cylinder	PE17, PE18, PE19	Mechanical
1200	180	Cylinder	PE14, PE15, PE16	Mechanical
1200	180	Round disk	PE30, PE31, PE32, PE33, PE34, PE35	In vitro
1300	90	Cylinder	PE4, PE5, PE6	Mechanical
1300	120	Cylinder	PE1, PE2, PE3	Mechanical
1300	180	Cylinder	PE7, PE8, PE9	Mechanical
1400	90	Cylinder	PE10, PE11, PE12	Mechanical
1400	120	Cylinder	PE23, PE24, PE25	Mechanical
1400	180	Cylinder	PE13, PE27, PE29	Mechanical

**Table 2:** Chemical analysis results.

Sample Name	Ca, Weight %	P, Weight %	Ca:P Ratio
HAP-20	38.4	17.1	1.74
HAP-60	40.3	17.0	1.84
HAP-100	39.5	17.4	1.76

**Table 3:** Values of absorbance ratios A_1097_/A_1487_ and A_962_/A_1421_ as obtained from the spectra of HAp nanopowders at different pressures.

Sample Name	A_1097_/A_1487_	A_962_/A_1421_
HAP-20	13.55	1
HAP-60	9	1
HAP-100	15.25	1.42

**Table 4:** Scherrer crystallite size, BET average particle size, and hydrodynamic diameter of HAP nanopowders.

Sample Name	Crystallite Size in (002) Direction, nm	CI, %	Average Particle Size (BET), nm	d (H), nm	PdI
HAP-20	28	71.1	18.7	76	0.009
HAP-60	33	77.8	21.7	84	0.087
HAP-100	38	79.4	37.3	97	0.085

**Table 5:** Elemental compositions of HAp nanopowders.

Element	Weight %		
	HAP-20	HAP-60	HAP-100
Ca K	27.49	34.41	32.83
P K	12.26	14.83	14.44
O K	37.64	42.65	44.57
C K	17.68	8.11	3.89
Au K	4.92	-	4.26
Ca: P ratio (EDS analysis)	1.74	1.79	1.78
Ca:P ratio (chemical analysis)	1.74	1.84	1.76

**Table 6:** The compressive strength of the specimens sintered at various sintering times.

Sintering Conditions	Compressive Strength, N/mm^2^		
	90 Min	120 Min	180 Min
1100 °C	37.65–39.13	-	-
1200 °C	49.08–102.24	49.79–76.28	64.54–127.15
1300 °C	20.21–76.93	58.81–86.12	26.72–73.81
1400 °C	37.00–65.81	75.55–120.23	51.17–206.40

**Table 7:** Wear test-measured values for coefficient of friction and maximum penetration.

**Sintering Temperature, °C**	1100	1200			1300			1400		
**Sintering Time, Minutes**	90	90	120	180	90	120	180	90	120	180
**Coefficient of Friction, µ**	0.032	0.031	0.031	0.031	0.031	0.031	0.031	0.031	0.031	0.031
**Maximum Depth, p, mm**	0.88	0.76	0.72	0.72	0.75	0.69	0.72	0.71	0.68	0.69

## Data Availability

Not applicable. The results of the study were not published in other journals, conferences, public databases, etc.

## Supplementary Materials

The following supporting information can be downloaded at: https://www.mdpi.com/article/10.3390/ma15155091/s1, Figure S1: Particle size distribution of: (a) HAP-20; (b) HAP-60 and (c) HAP-100 nanopowders; Figure S2: SEM images at 5 μm scale bar, 5 kV voltage and 20 kX magnification, and EDS spectra of HAp nanopowders: (a) and (b) HAP-20; (c) and (d) HAP-60.
