# Supplementary material for: Hydroxyapatite from Natural Sources for Medical Applications
Source: Materials (Basel). 2022 Jul 22;15(15):5091. doi: 10.3390/ma15155091 (PMC9331458; doi:10.3390/ma15155091)
Supplement: Supplementary file 1 [file materials-15-05091-s001.zip › materials-1819087-supplementary.pdf]

materials

MDPI

Hydroxyapatite from natural sources for medical applications

Laura Madalina Cursaru[1*], Miruna Iota[1], Roxana Mioara Piticescu[1*], Daniela Tarnita[2], Sorin Vasile Savu[3], Ionel Dănuț Savu[3], Gabriela Dumitrescu[4], Diana Mihaela Popescu[4], Radu-Gabriel Hertzog[4] and Mihaela Calin[1,5]

* Correspondence: mpopescu@imnr.ro (L.M.C.); roxana.piticescu@imnr.ro (R.M.P.)

Results

			Size (d.nm):	% Intensity:	St Dev (d.n...
Z-Average (d.nm):	76.01	Peak 1:	79.64	100.0	18.62
PdI:	0.009	Peak 2:	0.000	0.0	0.000
Intercept:	0.872	Peak 3:	0.000	0.0	0.000
Result quality :	Good				

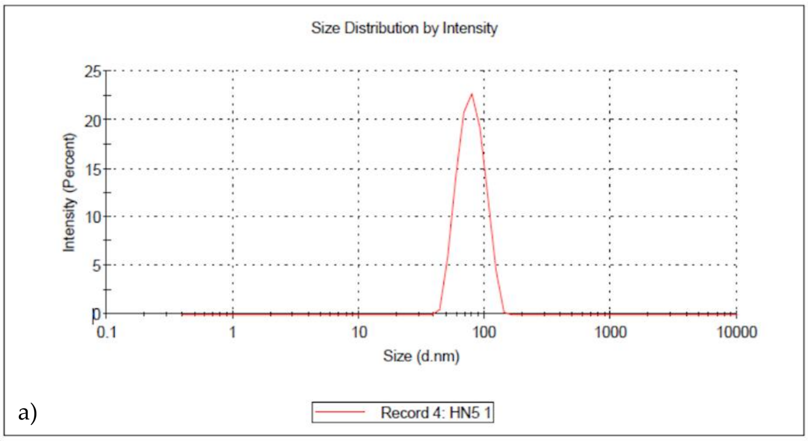

a)

Results

			Size (d.nm):	% Intensity:	St Dev (d.n...
Z-Average (d.nm):	84.11	Peak 1:	93.36	100.0	29.80
PdI:	0.087	Peak 2:	0.000	0.0	0.000
Intercept:	0.887	Peak 3:	0.000	0.0	0.000
Result quality :	Good				

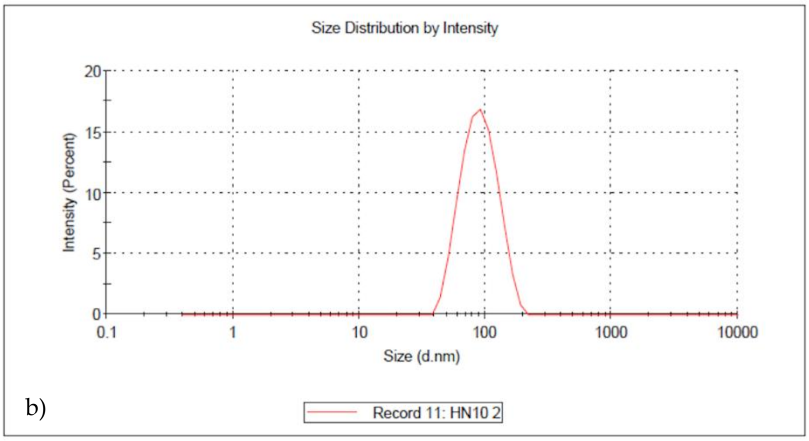

b)

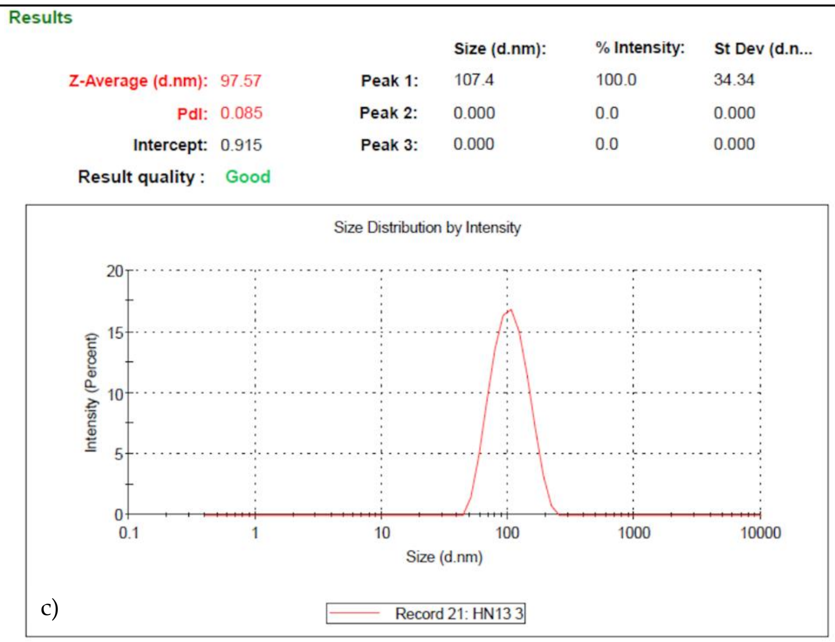

Figure S1. Particle size distribution of: a) HAP-20; b) HAP-60 and c) HAP-100 nanopowders.

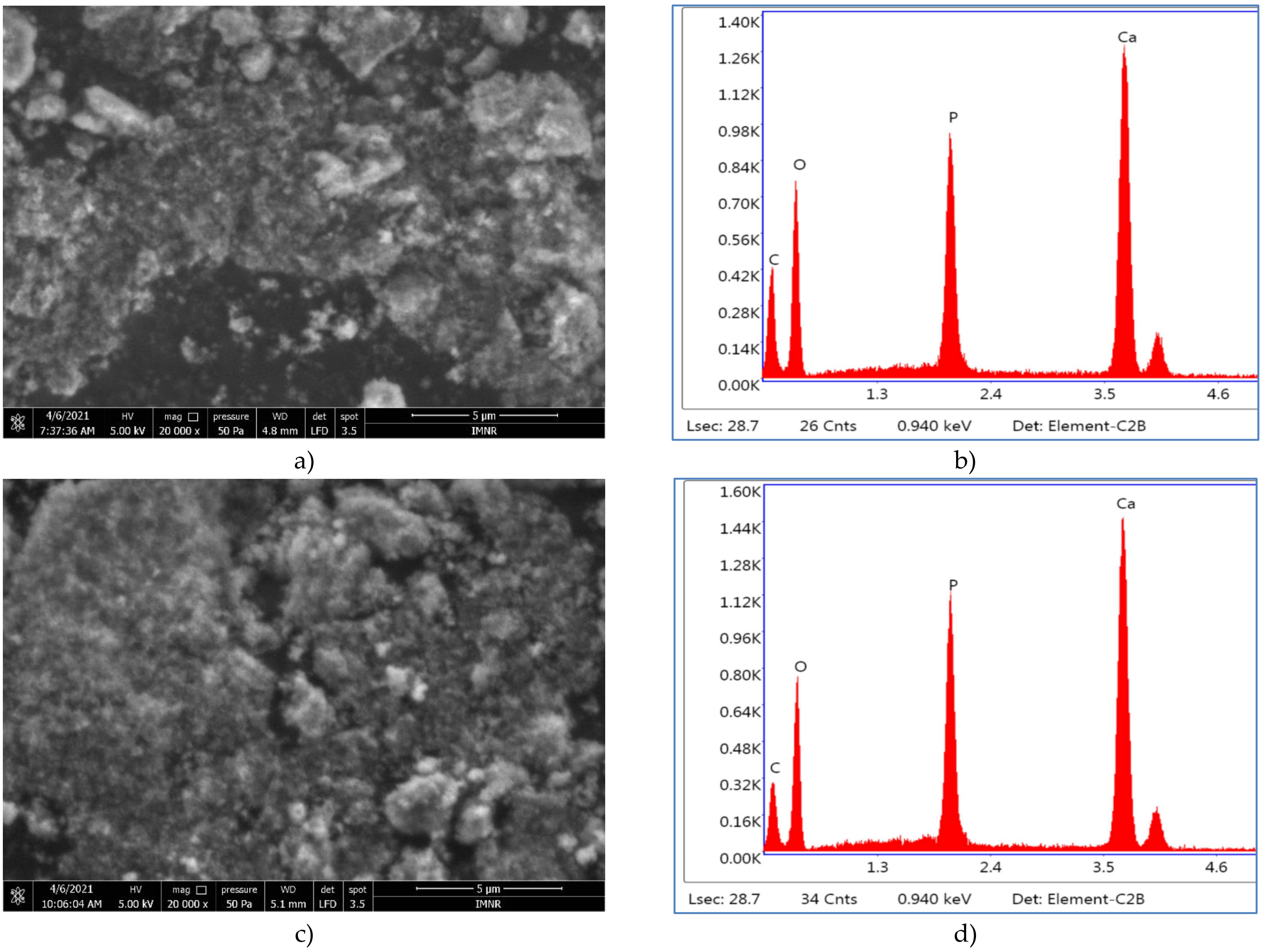

Figure S2. SEM images at 5 μm scale bar, 5 kV voltage and 20 kX magnification, and EDS spectra of HAp nanopowders: a) and b) HAP-20; c) and d) HAP-60.
